# Complete mitochondrial genome of *Trachycardium flavum* (Linnaeus, 1758)

**DOI:** 10.1080/23802359.2019.1624211

**Published:** 2019-07-11

**Authors:** Liyun Pu, Hongtao Liu, Mingqiu Yang, Guofu Wang, Guangyuan Xia, Minghui Shen, Bingshun Li

**Affiliations:** Hainan Provincial Engineering Research Center for Tropical Sea-farming, Hainan Academy of Ocean and Fisheries Sciences, Haikou, China

**Keywords:** Mitochondrial genome, *Trachycardium flavum*, phylogenetic tree

## Abstract

The complete mitochondrial genome of *Trachycardium flavum* from South China Sea was first determined in this study, which is 16,596 bp in length. The base composition of the mitogenome is a less biased (A, G, T, and C was 21.5%, 26.3%, 37.0%, and 15.2%, respectively) with A + T contents of 58%. It consists of 24 tRNA genes, two rRNA genes, and 12 protein-coding genes (PCGs). No typical control region was found in non-coding sequence. Interestingly, ATP8 genes was absence same as most shellfish. Nine PCGs use a special start codon TTG, and the other three genes use a normal initiation codon ATN. Except ND6 use a single base G as the stop codon, the others all end with the normal stop codon TAG. The phylogenetic tree showed that *T. flavum* was first clustered with *Fulvia mutica*, *Cerastoderma edule*, and *Acanthocardia tuberculate*, which all belong to Cardiidae.

*Trachycardium flavum,* which has been accepted as *Vasticardium flavum* in some marine taxonomic data, is a species in the family Cardiidae, Veneroida. It is widely distributed in the India-Western Pacific tropical region, mainly in Mainland China and Taiwan. It is often buried in the sandy bottom of the intertidal low tide line to the shallow sea, and feeds by filtering microalgae and organic debris. Due to its high economic value and edible value, many researchers have paid much attention to the biology of *T. flavum* for farming and breeding in recent years, including histology of gonad (Wu et al., 2004), main economic traits analysis (Guo, Chen, et al. 2013a), filtration rate (Guo, Wang, et al. 2013b), metabolic rates and immune-related enzymatic activities (Tang et al. 2010, 2017), ingestion and digestion of larvae(Deng et al. 2016).

The specimens were collected from Tanmen wharf of Qionghai, Hainan province, China (N19°14′12.12″, E110°37′17.36″) and stored in the Qionghai research base of Hainan Academy of Ocean and Fisheries Sciences. Muscle samples of *T. flavum* were preserved in absolute ethanol for total genomic DNA extraction.

The complete circular mitochondrial genome of *T. flavum* is 16,596 bp in length (GenBank Accession No. MK783266). It consists of 24 tRNA genes, two rRNA genes, 12 protein-coding genes (PCGs), and a non-coding region was atypical with 24,162 bp in length. Six overlap between adjacent genes were found varying from 1 bp to 23 bp. The nucleotide base content of *T. flavum* mitogenome was 21.5% A, 26.3% G, 37.0% T, and 15.2% C. the 58.5% of (A + T) showed a little preference to AT. All genes were encoded on the heavy strand.

24 tRNA genes of the *T. flavum* mitogenome vary from 63 bp to 73 bp in length. Four tRNA genes are present more than once: tRNA-Asn has three copies; tRNA-Ser, tRNA-Met, and tRNA-Leu each has two copies. Interestingly, tRNA-Ala is not found in the genome. The 12S rRNA is 899 bp in length and located between ND4 and ND6, and the 16S rRNA is 1244 bp in length, located between CYTB and ND1. Similar as some shellfish species, among the expected 13 PCGs, 12 were identified, and ATP8 gene was absence, same as most bivalve species (Kong et al. 2015). Nine PCGs use a special codon TTG as the initiation codon, except ND2 uses ATG, COX2 uses ATA, and ND4 uses ATT. 11 PCGs were terminated with a stop codon TAG, only ND6 was ended with an uncomplete stop codon with a single base G. None microsatellite was identified in the mitogenome using MISA.

The phylogenetic tree was constructed based on the mitochondrial genome sequence of 16 shellfish species in Veneroida, by maximum-likelihood (ML) method with 1000 bootstrap replicates. The result (Figure 1) showed that *T. flavum* was first clustered with *Fulvia mutica*, *Cerastoderma edule*, and *Acanthocardia tuberculate*, which all belong to Cardiidae.

**Figure 1.**
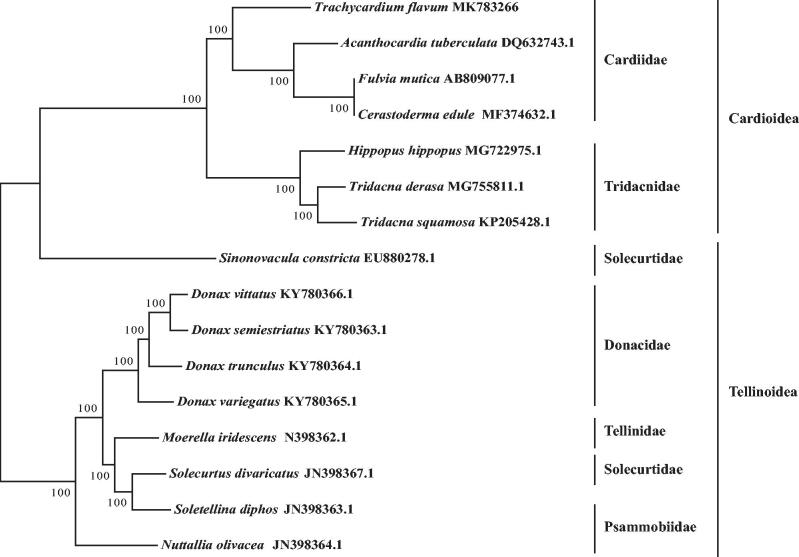
Phylogenetic tree based on 12 PCGs of 16 shellfish species mitochondrial genome by ML method.
